# Aharonov and Bohm versus Welsh eigenvalues

**DOI:** 10.1007/s11005-018-1069-9

**Published:** 2018-03-15

**Authors:** P. Exner, S. Kondej

**Affiliations:** 1 Doppler Institute for Mathematical Physics and Applied Mathematics, Czech Technical University in Prague, Břehová 7, 11519 Prague, Czechia; 2 Nuclear Physics Institute, Czech Academy of Sciences, 25068 Řež near Prague, Czechia; 3 Institute of Physics, University of Zielona Góra, ul. Szafrana 4a, 65246 Zielona Góra, Poland

**Keywords:** Singular Schrödinger operator, Radial symmetry, Discrete spectrum, Aharonov–Bohm flux, 81Q10, 35J10

## Abstract

We consider a class of two-dimensional Schrödinger operator with a singular interaction of the $\delta$ type and a fixed strength $\beta$ supported by an infinite family of concentric, equidistantly spaced circles, and discuss what happens below the essential spectrum when the system is amended by an Aharonov–Bohm flux $\alpha \in [0,\frac{1}{2}]$ in the center. It is shown that if $\beta \ne 0$, there is a critical value $\alpha _{\mathrm {crit}}\in (0,\frac{1}{2})$ such that the discrete spectrum has an accumulation point when $\alpha <\alpha _{\mathrm {crit}}$, while for $\alpha \ge \alpha _{\mathrm {crit}}$ the number of eigenvalues is at most finite, in particular, the discrete spectrum is empty for any fixed $\alpha \in (0,\frac{1}{2})$ and $|\beta |$ small enough.

## Introduction

Schrödinger operators with radially periodic potentials attracted attention because they exhibit interesting spectral properties. It was noted early [10] that the essential spectrum threshold of such an operator coincides with that of the one-dimensional Schrödinger operator describing the radial motion. More surprising appeared to be the structure of the essential spectrum which may consist of interlacing intervals of dense point and absolutely continuous nature as was first illustrated using potentials of cosine shape [11].

While this behavior can be observed in any dimension $\ge 2$, the two-dimensional case is of a particular interest because here these operators can also have a discrete spectrum below the threshold of the essential one. This fact was first observed in [5] and the national pride inspired the authors to refer to this spectrum as to *Welsh eigenvalues*; it was soon established that that their number is infinite if the radially symmetric potential is nonzero and belongs to $L^1_\mathrm {loc}\,$ [17]. Moreover, the effect persists if such a regular potential is replaced by a periodic array of $\delta$ interactions or more general singular interactions [7, 8].

The question addressed in this paper is how are the Welsh eigenvalues influenced by a local magnetic field preserving the rotational symmetry. For simplicity, we will choose the simplest setting, the two-dimensional system with $\delta$ potential of a fixed strength $\beta$ supported on a concentric family of circles $\{\mathcal {C}_{r_{n}}\}_{n\in \mathbb {N}}$ or radii $r_n=d(n+\frac{1}{2}),\, n=0,1,\dots \,$, with $d>0$. Without the presence of the magnetic field, the corresponding Hamiltonian can be symbolically written as

$$\begin{aligned} H_{\beta } = -\Delta +\beta \sum _{n}\delta (x-\mathcal {C}_{r_{n}}),\quad \beta \in \mathbb {R}, \end{aligned}$$

which can be given meaning as a self-adjoint operator in $L^2(\mathbb {R}^2)$ as we will recall below. As we have said, the discrete spectrum of $H_\beta$ is infinite [8], which is a direct consequence of the fact that the effective potential in the s-wave component contains the term $-\frac{1}{4r^2}$ producing an infinite number of eigenvalues below $\inf \sigma _\mathrm {ess}(H_\beta )$.

The magnetic interaction we add is also chosen in the simplest possible way, namely as an Aharonov–Bohm flux $\alpha$ at the origin of the coordinates, measured in suitable units, that gives rise to a magnetic field vanishing outside this point. The corresponding Hamiltonian will be denoted $H_{\alpha ,\beta }$ and as we will argue, it is sufficient to consider flux values up to half of the quantum, $\alpha \in (0,\frac{1}{2})$. Since singular interactions are involved, it may be useful to stress that we consider an Aharonov–Bohm flux alone, without any additional point interactions at origin *à la* [1, 6]. It is known that local magnetic fields generally, and Aharonov–Bohm fluxes in particular, can reduce the discrete spectrum, if combined with an effective potential that behaves like $r^{-2}$, on the borderline between short and long range, the effect can be dramatic [14].

We are going to show that in the present model the Aharonov–Bohm field also influences the discrete spectrum but the dependence on the flux value is more complicated. Specifically, we claim that

- there is an $\alpha _{\mathrm {crit}}(\beta )=\alpha _{\mathrm {crit}} \in (0, \frac{1}{2})$ such that for $\alpha \in (0,\alpha _{\mathrm {crit}})$ the discrete spectrum of $H_{\alpha ,\beta }$ is infinite accumulating at the threshold $E_0$, while for $\alpha \in [\alpha _{\mathrm {crit}},\frac{1}{2})$ there is at most a finite number of eigenvalues below $E_0$,
- the critical value $\alpha _{\mathrm {crit}}(\beta )$ admits the following asymptotics,
  $$\begin{aligned} \alpha _{\mathrm {crit}}(\beta )\rightarrow \frac{1}{2} - \quad \mathrm {for }\quad \beta \rightarrow \pm \infty \end{aligned}$$
  and
  $$\begin{aligned} \alpha _{\mathrm {crit}}(\beta )\rightarrow 0 + \quad \mathrm {for }\quad \beta \rightarrow 0 , \end{aligned}$$
- for any fixed $\alpha \in (0, \frac{1}{2} )$ there exists $\beta _0>0$ such that for any $|\beta |\le \beta _0$ we have $\sigma _\mathrm {d}(H_{\alpha ,\beta }) = \emptyset$, and moreover, $\sigma _\mathrm {d}(H_{\frac{1}{2},\beta }) = \emptyset$ holds for any $\beta \in \mathbb {R}$.

These properties will be demonstrated in Sects. 3 and 4; before coming to that, in the next section we introduce properly the Hamiltonian and derive its elementary properties.

## Preliminaries

We consider a magnetic flux $\phi$ perpendicular to the plane to which the particle is confined and placed at the origin of the coordinates corresponding to the vector potential

$$\begin{aligned} A(x,y) = \frac{\phi }{2\pi }\left( -\frac{y}{r^2}, \frac{x}{r^2} \right) . \end{aligned}$$

In the rational units we use, the flux quantum is $2\pi$, thus it is natural to introduce $\alpha := \frac{\phi }{2\pi }$. Given this *A* we define the ‘free’ Aharonov–Bohm Hamiltonian

$$\begin{aligned} H_\alpha := (-i\nabla - A)^2,\quad D(H_\alpha )= \left\{ f\in L^2 (\mathbb {R}^2):\, (-i\nabla - A)^2 f \in L^2 \right\} , \end{aligned}$$

where the domain is sometimes dubbed magnetic Sobolev space. Since the integer part of a given $\alpha$ can be removed by a simple gauge transformation, it is sufficient to consider $\alpha \in (0,1)$ only. The possibility of neglecting the integer part of $\alpha$ is also obvious from the partial wave decomposition presented below, cf. (2.1) and (2.2).

The radial symmetry allows us to describe $H_\alpha$ in terms of the partial wave decomposition. To this aim, we introduce the unitary operator $U\,:\, L^2 (\mathbb {R}_+, r\mathrm {d}r)\rightarrow L^2 (\mathbb {R}_+)$ acting as $Uf (r)= r^{1/2} f(r)$. This naturally leads to

$$\begin{aligned} L^{2} (\mathbb {R}^2 )= \bigoplus _{l\in \mathbb {Z}} U^{-1} L^2 (\mathbb {R}_+ ) \otimes S_l, \end{aligned}$$

where $S_l$ is the subspace spanned by $\mathrm {e}^{il\theta }$, the eigenspace of angular momentum operator on the unit circle, and the corresponding decomposition of the Hamiltonian

$$\begin{aligned} H_{\alpha }= \bigoplus _{l} U^{-1} H_{\alpha , l } U \otimes I_l, \end{aligned}$$

where $I_l$ is the identity operator on $S_l$ and the radial part is

2.1 $$\begin{aligned} H_{\alpha ,l}: =&- \frac{d^2}{d^2r} +\frac{1}{r^2}c_{\alpha ,l},\quad c_{\alpha ,l}:= -\frac{1}{4}+(l+\alpha )^2, \end{aligned}$$

2.2 $$\begin{aligned} D(H_{\alpha ,l}) :=&\,\left\{ f\in L^2 (\mathbb {R}_+) \,:\, -f''+\frac{ c_{\alpha , l} }{r^2}f\in L^2 (\mathbb {R}_+),\right. \nonumber \\&\quad \left. \lim _{r\rightarrow 0^+ }r^{\alpha -1/2}f(r)=0 \;\; \mathrm {if}\;\, l=0,\right. \nonumber \\&\quad \left. \lim _{r\rightarrow 0^+ }r^{1-\alpha -1/2}f(r)=0 \;\; \mathrm {if}\;\, l=-1 \right\} . \end{aligned}$$

We recall that this operator describes a ‘pure’ Aharonov–Bohm field without an additional singular interaction at the origin [1, 6]. This corresponds to the choice of $H_{\alpha , l},\, l=0,-1$, as appropriate self-adjoint extensions of the operator $- \frac{d^2}{d^2r} +\frac{1}{r^2}c_{\alpha , l}$ restricted to $C_0^\infty (\mathbb {R}_+)$. For all the other values of *l* the centrifugal term ensures the essential self-adjointness, here we choose the conditions which exclude the more singular of the two solutions at the origin, $r^{1/2}K_\alpha (\kappa r)$ and $r^{1/2}K_{1-\alpha } (\kappa r)$, respectively, where $K_\nu (\cdot )$ stands for the modified Bessel function of the second order, cf. [3, Eq. 9.6.23].

In the next step, we consider the $\delta$ *interaction supported by concentric circles*; we amend the system governed by $H_\alpha$ by a singular radially periodic potential supported by concentric circles $\mathcal {C}_{r_n}$ of the radii $r_n =d(n+\frac{1}{2})$, $\,d>0$, the strength of which is characterized by a nonzero coupling constant $\beta \in \mathbb {R}$. Since the radial symmetry is preserved, the resulting Hamiltonian can be again expressed in terms of its partial wave components,

2.3 $$\begin{aligned} H_{\alpha ; \beta } = \bigoplus _{l} U^{-1} H_{\alpha ;\beta , l } U \otimes I_l, \end{aligned}$$

where

2.4 $$\begin{aligned} D(H_{\alpha ;\beta ,l}) :=&\left\{ f \in W^{2, 2} (\mathbb {R}_+ \setminus \cup _{n\in \mathbb {N}} \{x_n\} ): \, f\, \mathrm {satisfies}\, (2.2)\right. \end{aligned}$$

2.5 $$\begin{aligned}&\left. \mathrm {and}\; \partial _r f(r_n^+)-\partial _r f(r_n^-) = \beta f(r_n ),\; n\in \mathbb {N}\right\} ; \end{aligned}$$

it is easy to check that operator $H_{\alpha ;\beta }$ is self-adjoint.

As in [7] it is useful to introduce a one-dimensional comparison operator which is the usual *Kronig–Penney Hamiltonian* with equidistantly spaced $\delta$ interactions supported by the set $\{x_n :=d(n+\frac{1}{2})\,:\, n\in \mathbb {Z}\}$. We denote it by $\mathrm {h}_\beta$, it acts as $\mathrm {h}_\beta f=-f''$ on the domain

$$\begin{aligned} D(\mathrm {h}_\beta ) =\left\{ f \in W^{2,2} (\mathbb {R}\setminus \cup _{n\in \mathbb {Z}}\{x_n \})\,:\, f'(x_n^+)- f'(x_n^-) = \beta f(x_n ),\; n\in \mathbb {Z}\right\} . \end{aligned}$$

Let $$E_0 $$ stand for the spectral threshold of $$\mathrm {h}_\beta $$,

2.6 $$\begin{aligned} E_0 := \inf \sigma (\mathrm {h}_\beta ); \end{aligned}$$

mimicking the argument used in [8] we can check easily that this quantity determines the essential spectrum of $H_{\alpha ; \beta }$, namely

2.7 $$\begin{aligned} \sigma _{\mathrm {ess}} (H_{\alpha ;\beta }) =[E_0,\infty ). \end{aligned}$$

Although it is not important for the present work, let us add that the reasoning made in [8] remains valid if the centrifugal coefficients in (2.1) replace their nonmagnetic values $c_{0,l}$, and consequently, the essential spectrum is not affected by the Aharonov–Bohm flux, consisting of the absolutely continuous bands that coincide with the spectral bands of $\mathrm {h}_\beta$ and the dense point part filling the spectral gaps of $\mathrm {h}_\beta$.

Our interest here concerns the spectrum of $H_{\alpha ;\beta }$ in the interval $(-\infty ,E_0)$ which is discrete according to (2.7). Let us first collect its elementary properties.

### Proposition 2.1

Suppose that $\beta \ne 0$, then

(i) $$\sharp \sigma _\mathrm {disc}(H_{0;\beta })=\infty $$;
(ii) $$\sigma _\mathrm {disc}(H_{\frac{1}{2};\beta })=\emptyset $$;
(iii) $$\sigma _\mathrm {disc}(H_{\alpha ;\beta })=\sigma _\mathrm {disc}(H_{1-\alpha ;\beta })$$;
(iv) if $\sigma _\mathrm {disc}(H_{\alpha ;\beta })\ne \emptyset$, then eigenvalues of $H_{\alpha ;\beta }$ are nondecreasing in $[0,\frac{1}{2}]$, $\,\lambda _j(\alpha ')\ge \lambda _j(\alpha )$ holds for a fixed *j* if $\alpha '\ge \alpha$.

### Proof

Claim (i) follows from [8, Theorem 5.1]. Partial wave operators in the decomposition (2.3) can contribute to $\sigma _\mathrm {disc}(H_{\alpha ;\beta })$ only if $c_{\alpha ,l}<0$. Indeed, if $c_{\alpha ,l}=0$ the spectrum of $H_{\alpha ;\beta ,l}$ coincides, up to multiplicity, with that of the operator $\mathrm {h}_\beta$ amended according to (2.2) with Dirichlet condition at $x=0$, hence (2.6) in combination with a bracketing argument [15, Sect. XIII.15] shows that the discrete spectrum is empty and yields assertion (ii). Furthermore, in view of the min-max principle [15, Sect. XIII.1] this verifies the above claim and shows that the discrete spectrum comes from $H_{\alpha ;\beta ,0}$ if $\alpha \in [0,\frac{1}{2})$ and from $H_{\alpha ;\beta ,-1}$ if $\alpha \in (\frac{1}{2},1)$. The third claim follows from the identity $c_{\alpha ,0}=c_{1-\alpha ,-1}$ valid for $\alpha \in (0,1)$, and the last one we get employing the min-max principle again. $\square$

It is therefore clear, as indicated in the introduction, that to describe the discrete spectrum it is sufficient to limit our attention to the values $\alpha \in (0,\frac{1}{2})$ and to consider the operator $H_{\alpha ;\beta ,0}$.

## Properties of the discrete spectrum

The previous discussion shows that the discrete spectrum for $\alpha \in (0,\frac{1}{2})$ comes from the partial wave operator $H_{\alpha ; \beta ,0}$ and the decisive quantity is the coefficient $c_{\alpha ,0}= \alpha ^2-\frac{1}{4}$. Let *y* be the solution of

3.1 $$\begin{aligned} H_{\alpha ;\beta ,0} g=E_0 g, \end{aligned}$$

where $E_0$ is the threshold value (2.6). We are going to employ the oscillation theory; following its general strategy, we introduce the Prüfer variables $(\rho , \theta )$ as follows

$$\begin{aligned} \left( \begin{array}{c} y \\ y' \end{array} \right) = \rho \left( \begin{array}{c} \cos \theta \\ \sin \theta \end{array} \right) . \end{aligned}$$

As it is usually the case with singular potentials [8], we can rephrase the discrete spectrum analysis as investigation of the asymptotic behavior of the function $r\mapsto \theta (r)$; for the reader’s convenience, the needed facts from the oscillation theory are collected in Sect. 5.

To formulate the first main result, we denote by *u* the *d*-periodic real-valued solution of the one-dimensional comparison problem,

3.2 $$\begin{aligned} {h}_\beta u=E_0 u. \end{aligned}$$

Then we can make the following claim.

### Theorem 3.1

Suppose that $\alpha \in (0,\frac{1}{2})$ and put

$$\begin{aligned} c_{\mathrm {crit}}:= - \frac{1}{4} \left( \frac{1}{d}\int _{0}^d \frac{1}{u^2}\,\mathrm {d}x\right) ^{-1} \left( \frac{1}{d} \int _{0}^d u^2\,\mathrm {d}x\right) ^{-1}. \end{aligned}$$

Then $E_0$ is an accumulation point of $\sigma _\mathrm {disc}(H_{\alpha ; \beta ,0})$ provided $\frac{c_{\alpha ,0}}{c_{\mathrm {crit}}} >1$, while for $\frac{c_{\alpha ,0}}{c_{\mathrm {crit}}} \le 1$ the operator has at most finite number of eigenvalues below $E_0$ with the multiplicity taken into account.

### Proof

The asymptotic properties of the function $\theta$ can be found in a way similar to that used in [18]. Let $u,\,v$ be linearly independent real-valued solutions of equation (3.2), where *u* is the mentioned positive *d*-periodic function involved in the definition of $c_{\mathrm {crit}}$, chosen in such a way that the Wronskian $W[u,v]=1$. Furthermore, we introduce the generalized Prüfer variables

3.3 $$\begin{aligned} \left( \begin{array}{c} y \\ y' \\ \end{array} \right) = \left( \begin{array}{cc} u &{} v \\ u' &{} v' \\ \end{array} \right) a \left( \begin{array}{c} \sin \gamma \\ -\cos \gamma \end{array} \right) , \end{aligned}$$

where *a* is a smooth positive function and $\gamma$ is continuous in view of [8, Lemma 3.4]. On the other hand, by [18, Proposition 1] the functions $\gamma (\cdot )$ and $\theta (\cdot )$ have the same asymptotics up to the constant. Consequently, it is sufficient to investigate the asymptotics of $\gamma (\cdot )$ which we will do using the expression

$$\begin{aligned} \gamma ' = \frac{c_{\alpha ,0}}{r^2}\left( u\sin \gamma -v \cos \gamma \right) ^{2}= c_{\alpha ,0}\,u^2\,\cos ^2 \gamma \, \left( \frac{1}{r}\tan \gamma -\frac{v}{ru}\right) ^2, \end{aligned}$$

which can be obtained from (3.3) by a direct computation using (3.1) and the Wronskian properties of the functions *u*, *v*. In the next step, we employ the Kepler transformation

$$\begin{aligned} \tan \phi = \frac{1}{r}\, \tan \gamma -\frac{1}{r}\frac{v}{u}, \end{aligned}$$

which yields

3.4 $$\begin{aligned} \phi ' = \frac{1}{r} \left( -\sin \phi \cos \phi +\mathcal {B}(r) \sin ^2 \phi + \mathcal {A}(r) \cos ^2 \phi \right) , \end{aligned}$$

where $\mathcal {A}$ and $\mathcal {B}$ are the *d*-periodic functions defined by

3.5 $$\begin{aligned} \mathcal {B}(r):=c_{\alpha ,0} u(r)^2 \quad \; \mathrm {and } \quad \; \mathcal {A}(r):= - \frac{1}{u(r)^2}. \end{aligned}$$

The Kepler transformation preserves the asymptotics, i.e., $\gamma (r)= \phi (r)+\mathcal {O}(1)$ holds as $r\rightarrow \infty$, thus we may inspect the asymptotics of $\phi (\cdot )$. This can be done in the same way as for regular period potentials. Specifically, we define

3.6 $$\begin{aligned} {\overline{\phi }} (r):=\frac{1}{d }\int _{r}^{r+d}\phi (\xi )\,\mathrm {d}\xi , \quad r>R_0, \end{aligned}$$

for some $R_0>0$. Proposition 2 of [18] allows us to conclude that ${\overline{\phi }}(r)=\phi (r)+o(1)$ and

3.7 $$\begin{aligned} {\overline{\phi }}'(r) = \frac{1}{r} \left( -\sin {\overline{\phi }} \cos {\overline{\phi }} + B \sin ^2 {\overline{\phi }} + A \cos ^2 {\overline{\phi }} \right) +\mathcal {O}(r^{-2}), \end{aligned}$$

where

$$\begin{aligned} A:=\frac{1}{d}\int _{0}^d \mathcal {A}(r)\,\mathrm {d}r \quad \; \mathrm {and } \quad \; B:=\frac{1}{d}\int _{0}^d \mathcal {B}(r)\,\mathrm {d}r. \end{aligned}$$

Now we apply Proposition 3 of [18] which states that ${\overline{\phi }}$ is bounded provided $4AB<1$ and unbounded if $4AB>1$. Combining this fact with the observation that

$$\begin{aligned} 4AB = -4c_{\alpha , 0} \left( \frac{1}{d }\int _{0}^d \frac{1}{u^2}\,\mathrm {d}r\right) \left( \frac{1}{d }\int _{0}^d u^2\,\mathrm {d}r\right) = \frac{c_{\alpha ,0}}{c_{\mathrm {crit}}} \end{aligned}$$

we come to the claim of the theorem for any $c_{\alpha , 0}$ apart from the case $c_{\alpha , 0} = c_{\mathrm {crit}}$. To complete the proof, we note that

$$\begin{aligned} \lim _{r\rightarrow 0 }\,r(\log r )^2 \left( {\overline{\phi }}'(r) - \frac{1}{r} \left( -\sin {\overline{\phi }} \cos {\overline{\phi }} + B \sin ^2 {\overline{\phi }} + A \cos ^2 {\overline{\phi }} \right) \right) =0 , \end{aligned}$$

cf. (3.7). Applying now Proposition 4 of [18], we conclude that if $4AB =1$ then ${\overline{\phi }}$ is globally bounded. This equivalently means that for $c_{\alpha , 0} = c_{\mathrm {crit}}$ at most finite number of discrete spectrum below $E_0$ can exist. $\square$

We note that the analogue of $c_{\mathrm {crit}}$ for regular potentials is known in the literature as Knesser constant, cf. [16]. The obtained result allows us to prove the following claim.

### Theorem 3.2

There exists an $\alpha _{\mathrm {crit}}(\beta )=\alpha _{\mathrm {crit}}\in (0,\frac{1}{2})$ such that for $\alpha \in (0, \alpha _{\mathrm {crit}})$ the operator $H_{\alpha , \beta }$ has infinitely many eigenvalues accumulating at the threshold $E_0$, the multiplicity taken into account, while for $\alpha \in [\alpha _{\mathrm {crit}},\frac{1}{2})$ the cardinality of the discrete spectrum is finite.

### Proof

The function $\alpha \mapsto c_{\alpha , 0} =\alpha ^2 -\frac{1}{4}$ is increasing in $(0,\frac{1}{2})$. Thus it suffices to show that $c_{\mathrm {crit}}\in (-\frac{1}{4},0)$ which is an easy consequence of the Schwartz inequality,

$$\begin{aligned} c_{\mathrm {crit}}:= - \frac{1}{4} \left( \frac{1}{d}\int _{0}^d \frac{1}{u^2}\,\mathrm {d}x\right) ^{-1} \left( \frac{1}{d} \int _{0}^d u^2\,\mathrm {d}x\right) ^{-1} > - \frac{1}{4} \left( \frac{1}{d }\int _{0}^d \mathrm {d}x\right) ^{-2} = - \frac{1}{4}\,; \end{aligned}$$

note that the inequality is sharp because the function *u* is nonconstant. The claim then follows from Theorem 3.1 if we set $\alpha _{\mathrm {crit}}:= \sqrt{c_{\mathrm {crit}}+\frac{1}{4}}$. $\square$

Moreover, in our present case the critical value can be computed explicitly because we know the function *u*, which is equal to

3.8 $$\begin{aligned} u(x) = \left\{ \begin{array}{ll} \mathrm {e}^{-\kappa _0 (x-d/2) }+\mathrm {e}^{\kappa _0 d}\mathrm {e}^{\kappa _0 (x-d/2)} &{} \quad \hbox {for }\,\, 0<x<\frac{d}{2}, \\ \mathrm {e}^{\kappa _0 d} \mathrm {e}^{-\kappa _0 (x-d/2)}+ \mathrm {e}^{\kappa _0 (x-d/2)} &{} \quad \hbox {for }\,\, \frac{d}{2}<x<d, \end{array} \right. \end{aligned}$$

cf. [2, Sect. III.2.3], where $i\kappa _0 = k_0$, $k_0^2=E_0$ comes from solution of the Kronig–Penney condition, Eq. (2.3.16) in [2], with $\theta =0$. While one cannot write the solution $\kappa _0=\kappa _0(\beta )$ in a closed form, its behavior is well known, cf. Theorem 3.2.3 and Figure 39 in [2]. Note further that the function (3.8) is obviously real-valued if $\beta <0$ so that $E_0<0$ and $\kappa _0>0$, in the opposite case with $\beta >0$ we have $E_0>0$ and $\kappa _0$ is purely imaginary, nevertheless *u* is a multiple of a real-valued function again. A straightforward calculation then yields

$$\begin{aligned} D_1: = \frac{1}{d }\int _{0}^d u^2\,\mathrm {d}x =\frac{2}{d}\,\mathrm {e}^{\kappa _0 d} \left( \frac{1}{2\kappa _0 } \left( \mathrm {e}^{\kappa _0 d }- \mathrm {e}^{-\kappa _0 d }\right) +d\right) \end{aligned}$$

and

$$\begin{aligned} D_2: = \frac{1}{d }\int _{0}^d \frac{1}{u^{2}}\,\mathrm {d}x =\frac{1}{d\kappa _0} \,\mathrm {e}^{-\kappa _0 d}\left( \frac{1}{2} - \frac{1}{1+\mathrm {e}^{\kappa _0 d }}\right) . \end{aligned}$$

Using this notation, we have

3.9 $$\begin{aligned} c_{\mathrm {crit}}=-\frac{1}{4} \frac{1}{D_1 D_2} = -\frac{1}{4} \kappa _0 d \left( \frac{\sinh (\kappa _0 d)}{\kappa _0 d} +1\right) ^{-1} \coth \left( \frac{\kappa _0 d }{2}\right) . \end{aligned}$$

These expressions allow us, in particular, to find the behavior of the critical flux values in the asymptotic regimes.

First, let us consider *weak coupling constant case*, $$\beta \rightarrow 0 $$. We derive the asymptotics of $$E_0 $$ on $$\beta $$ relying on the spectral conditions, cf. [2], Eqs. (2.3.24) and (2.3.25),

3.10 $$\begin{aligned} \coth \Big (\frac{1}{2}\kappa d\Big )=\frac{2\kappa }{|\beta |} \qquad \mathrm {for}\quad \beta <0\, \end{aligned}$$

and

3.11 $$\begin{aligned} \cot \Big (\frac{1}{2} kd\Big ) = \frac{2k}{\beta }\qquad \mathrm {for}\quad \beta >0. \end{aligned}$$

Then, for $\beta <0$ we have $E_0=-\kappa _0^2$ where $\kappa _0$ is the solution of (3.10) and for $\beta >0$ we have $E_0 =k_0^2$ where $k_0$ is the lowest solution of (3.11). This implies

$$\begin{aligned} E_0 = \frac{\beta }{d} -\frac{1}{12}\beta ^2+\mathcal {O} (\beta ^3), \end{aligned}$$

for $\beta \rightarrow 0$. Consequently, applying (3.9) one obtains

$$\begin{aligned} c_{\mathrm {crit}}= -\frac{1}{4} + \mathcal {O} (\beta ^2). \end{aligned}$$

This yields

3.12 $$\begin{aligned} \alpha _{\mathrm {crit}}(\beta ) = \mathcal {O} (\beta ^2), \quad \beta \rightarrow 0 \end{aligned}$$

which is certainly not surprising in view of the fact that the discrete spectrum is empty for $\beta =0$.

On the other hand, in the *strong coupling constant case* one has to take the sign of $\beta$ into account. We again apply (3.10) and (3.11) which this time give the asymptotics

$$\begin{aligned} E_0 = -\frac{\beta ^2}{4}\left( 1+ 4 e^{-|\beta |d/2}+ \mathcal {O}(e^{-|\beta | d}) \right) ,\qquad \beta \rightarrow -\infty \, \end{aligned}$$

and

$$\begin{aligned} E_0 = \left( \frac{\pi }{d}\right) ^2 - \frac{8\pi ^2}{\beta d^3} + \mathcal {O}(\beta ^{-2}), \qquad \beta \rightarrow \infty . \end{aligned}$$

Combining the above asymptotics with (3.9), we state that $c_{\mathrm {crit}}$ tends to zero, exponentially fast for the attractive $\delta$ interactions, i.e.,

$$\begin{aligned} c_{\mathrm {crit}} \approx -\frac{(d\beta ) ^2}{8}\, \mathrm {e}^{-|\beta | d/2}\, ,\qquad \beta \rightarrow -\infty \end{aligned}$$

and for the repulsive potential we have

$$\begin{aligned} c_{\mathrm {crit}} \approx - \frac{\pi ^2}{2\beta d} ,\qquad \beta \rightarrow \infty . \end{aligned}$$

Furthermore, this yields

3.13 $$\begin{aligned} \alpha _{\mathrm {crit}}(\beta ) = \frac{1}{2} + \mathcal {O} (\beta ^2 \mathrm {e}^{-|\beta | d/2}) \;\quad \mathrm {as } \quad \beta \rightarrow -\infty \end{aligned}$$

and

3.14 $$\begin{aligned} \alpha _{\mathrm {crit}}(\beta ) = \frac{1}{2} + \mathcal {O} (\beta ^{-1} ) \;\quad \mathrm {as } \quad \beta \rightarrow \infty . \end{aligned}$$

Hence the critical value is in the strong coupling regime close to $\frac{1}{2}$, the sign of $\beta$ shows up only in the error term.

## Nonexistence of the discrete spectrum for weak $\delta$ interactions

The above results tell us nothing about the spectrum of $H_{\alpha ; \beta }$ for $\alpha \in [\alpha _{\mathrm {crit}},\frac{1}{2})$, in particular, we do not know whether the operator may have some eigenvalues. Our aim now is to show that for a fixed $\alpha$, with the exception of the nonmagnetic and half-of-the-quantum cases, we have

$$\begin{aligned} \sigma _{\mathrm {disc}} (H_{\alpha ;\beta }) = \emptyset \end{aligned}$$

provided the involved $\delta$ interaction is sufficiently weak. Using a modified version of the Hardy inequality, we are going to prove the following claim:

### Theorem 4.1

Given $\alpha \in (0, \frac{1}{2} )$ there exists a $\beta _0 >0$ such that for any $|\beta | <\beta _0$ the operator $H_{\alpha ; \beta }$ has no discrete spectrum.

### Proof

To show that the discrete spectrum is void, it suffices to investigate the ‘lowest’ partial wave component $H_{\alpha ; \beta , 0}$. Consider the quadratic form associated with the ‘shifted’ operator $H_{\alpha ; \beta , 0}-E_0$,

4.1 $$\begin{aligned}&q_{\alpha ; \beta , 0} [f] \nonumber \\&\quad :=\int _{0}^\infty |f(r)'|^2\,\mathrm {d} r + c_{\alpha , 0 }\int _{0}^\infty \frac{1}{r^2}|f(r)|^2 \,\mathrm {d} r + \beta \sum _{n}\int _{\mathcal {C }_{r_n }} |f (r)|^2\,\mathrm {d} \mu _{{ \mathcal {C} _{r_n }}} - E_0 \Vert f\Vert ^2, \end{aligned}$$

where $\mu _{ \mathcal {C} _{r_n } }$ defines the arc length measure on $\mathcal {C} _{r_n }$ and $c_{\alpha , 0}\in (-1/4,0)$, moreover, $f\in D(H_{\alpha ; \beta , 0})$, i.e., it satisfies the boundary conditions given by (2.4) and (2.5). Without loss of generality, we may assume that *f* is a real function. As in the previous discussion, *u* stands for the periodic function defining the ‘lowest’ generalized eigenfunction of $\mathrm {h}_\beta$. We may assume that *u* is positive, then from the explicit expression (3.8) we see that for a fixed $\beta _1 >0$ there exists a $C_{\mathrm {min}}>0$ such that $u \ge C_{\mathrm {min}}$ holds for any $|\beta |\le \beta _1$. Furthermore, we put $\chi =\frac{f}{u}$; one can easily check that $\chi \in H_0^{2,2} (\mathbb {R}_+)$. Integrating by parts and using the boundary conditions (2.4) and (2.5) we get

$$\begin{aligned} q_{\alpha ;\beta ,0}[u\chi ] = -\int _{0}^\infty u \chi (u \chi )'' \,\mathrm {d}r+ c_{\alpha , 0}\int _{0}^\infty u^2\frac{\chi ^2}{r^2} \,\mathrm {d}r - E_0 \Vert u \chi \Vert ^2. \end{aligned}$$

After expanding the second derivative and using the equation that *u* as a generalized eigenfunction satisfies we get

4.2 $$\begin{aligned} q_{\alpha ;\beta ,0}[u\chi ]= & {} \int _{0}^\infty u ^2 \left( -\chi \chi '' + \frac{ c_{\alpha ,0} }{r^2}\chi ^2 \right) \,\mathrm {d}r - \int _{0}^\infty (u^2 )' \chi \chi ' \,\mathrm {d}r\, \nonumber \nonumber \\= & {} \int _{0}^\infty u ^2 (\chi ')^2 \,\mathrm {d}r + c_{\alpha ,0} \int _{0}^\infty u^2 \frac{ \chi ^2 }{r^2} \,\mathrm {d}r , \end{aligned}$$

where in the second step we performed integration by parts in the last expression of the first line with the boundary term vanishing due to (2.2). The following lemma will be useful in the further discussion. $\square$

### Lemma 4.2

We have

4.3 $$\begin{aligned} q_{\alpha ;\beta ,0}[u\chi ]> \alpha ^2 \int _{0}^\infty u^2 \frac{ \chi ^2 }{r^2}\,\mathrm {d}r - \frac{1}{2} \int _{0}^\infty (u^2)' \frac{ \chi ^2 }{r} \,\mathrm {d}r. \end{aligned}$$

### Proof

To prove the claim, we start from the expression

4.4 $$\begin{aligned} \int _{0}^\infty u^2 (( r^{-1/2 }\chi )' )^2 \,r \mathrm {d}r= \int _{0}^\infty u^2 \left( -\frac{1}{2} r^{-3/2 }\chi + r^{-1/2 }\chi ' \right) ^2 r \mathrm {d}r \nonumber \nonumber \\ = \int _{0}^\infty u^2 \left( \frac{1}{4r^2}\chi ^2 -\frac{1}{r} \chi \chi ' + (\chi ')^2 \right) \mathrm {d}r. \end{aligned}$$

On the other hand, the second term in (4.4) can be rewritten as

$$\begin{aligned} -\int _{0}^\infty u^2 \frac{\chi \chi '}{r}\,\mathrm {d}r =-\frac{1}{2} \int _{0}^\infty u^2 \frac{(\chi ^2)'}{ r}\mathrm {d}r = \frac{1}{2} \int _{0}^\infty \left( \frac{(u^2)' }{r} - \frac{u^2}{ r^2}\right) \chi ^2 \,\mathrm {d}r, \end{aligned}$$

where we have again employed integration by parts in combination with (2.2); inserting this to (4.4) we get

$$\begin{aligned} \int _{0}^\infty u^2 (( r^{-1/2 }\chi )' )^2 \,r \mathrm {d}r = \int _{0}^\infty u^2 \left( (\chi ')^2-\frac{\chi ^2}{4r^2} \right) \,\mathrm {d}r + \frac{1}{2} \int _{0}^\infty \frac{(u^2)' }{r} \chi ^2 \,\mathrm {d}r. \end{aligned}$$

Since $\int _{0}^\infty u^2 (( r^{-1/2 }\chi )' )^2 r \mathrm {d}r>0$, taking into account expression (4.2) and using $c_{\alpha , 0}= \alpha ^2 -\frac{1}{4}$ we obtain the claim of lemma. $\square$

With a further purpose in mind, we introduce a symbol for the second term at the right-hand side of (4.3),

$$\begin{aligned} \tilde{q}[\chi ] := -\frac{1}{2} \int _{0}^\infty (u^2)'\, \frac{\chi ^2 }{r} \,\mathrm {d}r. \end{aligned}$$

Our next aim is to show that $\tilde{q}[\cdot ]$ is small with respect to $q_{\alpha ; \beta , 0}[\cdot ]$. This is the content of the following lemma.

### Lemma 4.3

We have

4.5 $$\begin{aligned} |\tilde{q}[\chi ] | \le \eta (\beta ) \int _{0}^{\infty } (\chi ')^2 \,\mathrm {d}r, \end{aligned}$$

where the function $\eta (\cdot )$ behaves asymptotically as

$$\begin{aligned} \eta (\beta )=\mathcal {O}(\kappa _0 (\beta )) \end{aligned}$$

for $\beta$ small. Here $\kappa _0 = \kappa _0 (\beta )$ is the quantity introduced in (3.8) and the expression on the right-hand side does not depend on $\chi$.

### Proof

Note first that an integration by parts in combination with conditions (2.2) yields

$$\begin{aligned} \tilde{q}[\chi ]&= \frac{1}{2} \int _{0}^\infty u^2 \left( \frac{\chi ^2 }{r}\right) ' \mathrm {d}r= \frac{1}{2} \int _{0}^\infty \left( u^2(r)-u^{2}(0)\right) \left( \frac{\chi ^2 }{r}\right) ' \mathrm {d}r\\&=\frac{1}{2} \int _{0}^\infty \left( u^2(r)-u^{2}(0)\right) \left( \frac{2\chi \chi ' }{r} - \frac{\chi ^2}{r^2}\right) \mathrm {d}r. \end{aligned}$$

On the other hand, from the explicit expression (3.8) we get easily

$$\begin{aligned} \left| u^2(r)-u^{2}(0)\right| = \mathcal {O}(\kappa _0 (\beta )) \end{aligned}$$

as $\beta \rightarrow 0$ where the right-hand side does not depend on *r* since the function *u* is periodic, and naturally neither on $\chi$. Consequently,

4.6 $$\begin{aligned} |\tilde{q}[\chi ] | \le \eta _1 (\beta )\left( \int _{0}^\infty \frac{|2\chi \chi '| }{r} \,\mathrm {d}r + \int _{0}^\infty \frac{(\chi )^2 }{r^2} \,\mathrm {d}r \right) , \end{aligned}$$

where $\eta _1 (\beta )$ behaves asymptotically as $\eta _1 (\beta )= \mathcal {O}(\kappa _0 (\beta ))$. Our next aim is to estimate the first integral on the right-hand side of (4.6),

$$\begin{aligned} \tilde{q}_1[\chi ] := 2 \int _{0}^\infty \frac{|\chi \chi '| }{r} \,\mathrm {d}r. \end{aligned}$$

Applying the Schwartz inequality together with the classical Hardy inequality,

$$\begin{aligned} \int _{0}^\infty (\chi ')^2 \,\mathrm {d}r > \frac{1}{4} \int _{0}^\infty \frac{ \chi ^2 }{r^2} \,\mathrm {d}r, \end{aligned}$$

one obtains

$$\begin{aligned}&{\tilde{q}_1[\chi ] \le 2 \left( \int _{0}^\infty \frac{(\chi )^2 }{r^2} \,\mathrm {d}r \right) ^{1/2} \left( \int _{0}^\infty (\chi ' )^2 \,\mathrm {d}r \right) ^{1/2}} \\&\qquad \quad \le \int _{0}^\infty \frac{(\chi )^2 }{r^2} \,\mathrm {d}r + \int _{0}^\infty (\chi ' )^2 \,\mathrm {d}r < 5 \int _{0}^\infty (\chi ' )^2 \,\mathrm {d}r. \end{aligned}$$

Applying the Hardy inequality again to (4.6) and combining this with the above result we get

$$\begin{aligned} |\tilde{q}[\chi ] | \le \eta (\beta ) \int _{0}^\infty (\chi ' )^2 \,\mathrm {d}r, \end{aligned}$$

where $\eta (\beta ):= 9 \eta _1 (\beta )$. This completes the proof of lemma. $\square$

### Proof of Theorem 4.1, continued

As we noted above, for any $\beta$ satisfying $|\beta |\le \beta _1$ we have $\min _{r\ge 0} u(r)\ge C_\mathrm {min}$. Then the above lemma tells us that

4.7 $$\begin{aligned} |\tilde{q}[\chi ] | \le \eta (\beta ) \frac{1}{C_\mathrm {min}}\int _{0}^\infty u^2 (\chi ' )^2 \,\mathrm {d}r, \end{aligned}$$

which implies

4.8 $$\begin{aligned} \left| \tilde{q}[\chi ]\right| \le {\tilde{\eta }} (\beta ) \int _{0}^\infty u ^2 (\chi ')^2 \mathrm {d}r \quad \text {with}\quad {\tilde{\eta }} (\beta ) = \mathcal {O}(\kappa _0 (\beta )). \end{aligned}$$

On the other hand, by Lemma 4.2 we have

4.9 $$\begin{aligned} q_{\alpha ;\beta ,0}[u\chi ] > \alpha ^2 \int _{0}^\infty u^2 \frac{ \chi ^2 }{r^2} \mathrm {d}r+ \tilde{q}[\chi ]. \end{aligned}$$

Combining relations (4.8) and (4.9) we get

$$\begin{aligned} \left( 1+{\tilde{\eta }}(\beta )\right) \left( \int _{0}^\infty u ^2 (\chi ')^2 \,\mathrm {d}r + c_{\alpha ,0} \int _{0}^\infty u^2 \frac{ \chi ^2 }{r^2}\,\mathrm {d}r \right) > \left( \alpha ^2 + c_{\alpha , 0} {\tilde{\eta }}(\beta ) \right) \int _{0}^\infty u^2 \frac{ \chi ^2 }{r^2} \,\mathrm {d}r \end{aligned}$$

which implies

$$\begin{aligned} q_{\alpha ;\beta ,0}[u\chi ] > \frac{\alpha ^2 + c_{\alpha , 0} {\tilde{\eta }}(\beta )}{1+{\tilde{\eta }}(\beta )}\, \int _{0}^\infty u^2 \frac{ \chi ^2 }{r^2} \,\mathrm {d}r. \end{aligned}$$

By Lemma 4.3 there is a $\beta _0\in (0,\beta _1)$ such that for any $\beta$ satisfying $|\beta |\le \beta _0$ the pre-integral factor in the last formula is positive which means that we have

$$\begin{aligned} (H_{\alpha ; \beta , 0 }f,f )- E_0 \Vert f\Vert ^2 >0\, \end{aligned}$$

for any real function $f\in D(H_{\alpha ; \beta , 0 } )$. The same holds *mutatis mutandis* for the full Hamiltonian $H_{\alpha ; \beta }$ which completes the proof. $\square$

## Oscillation theory tools

To make the paper self-contained, we collect in this section the needed results of oscillation theory for singular potentials derived in [8]. Note that they extend the theory of Wronskian zeros for regular potentials developed in [9], related results can also be found in [19].

Consider points interaction localized at $x_n \in (l_-, l_+)$, where $n\in M \subset \mathbb {N}$. Moreover, assume that $q\in L^1_{\mathrm {loc} } (l_-, l_+)$ and combine the singular and regular potential in the operator on $L^2(l_-, l_+)$ acting as

$$\begin{aligned} Tu(x)= -u''(x)+q(x)u(x), \end{aligned}$$

with the domain

$$\begin{aligned}&D(T) := \left\{ f, f,\in AC_{\mathrm {loc}} (l_-, l_+) \setminus \left\{ x_n \,:\, n \in M \right\} \,:\right. \\&\left. Tu\in L^2 (l_-, l_+),\,\, \partial _r f(x_n^+)-\partial _r f(x_n^-) = \beta f(x_n ),\; n\in M \right\} . \end{aligned}$$

In general, the operator *T* is symmetric and we denote by *H* its self-adjoint extension satisfying either one of the following conditions

- *T* is limit point in at least one endpoint $l_\pm$
- *H* is defined by separated boundary conditions at the endpoints.

Suppose that there exist $\psi _\pm$ that satisfy the boundary conditions defining *H* at $l_\pm$ and $T\psi _\pm =E\psi _\pm$. Furthermore, let $W_0 (u_1, u_2)$ stand for the number of zeros of the Wronskian $W (u_1, u_2)= u_1u_2'-u_1'u_2$ in $(l_-, l_+)$ and denote $N_0 (E_1, E_2) := \mathrm {dim}\, \mathrm {Ran} P_{(E_1, E_2)}$, where $E_1 < E_2$ and $P_{(E_1, E_2)}$ is the corresponding spectral measure of *H*. Then we have [8]

5.1 $$\begin{aligned} W_0 (\psi _- (E_1),\psi _+ (E_2) ) = N_0 (E_1, E_2). \end{aligned}$$

In particular, the above equivalence allows us to estimate the cardinality of the discrete spectrum below the essential spectrum threshold $E_0$. Indeed, suppose $E<E_0$. Then, in the same way as for regular potentials, there exist $u=\psi _\pm (E)$ with the corresponding Prüfer angle $\theta$ bounded for *E* large negative. Expressing the Wronskian in the terms of the Prüfer variables $W[\psi _-(E),\psi _+(E_0) ]= \rho (x)\rho _0(x) \sin (\theta _0 (x)-\theta (x))$, we come to the conclusion that *the number of discrete spectrum points of* *H* *below* $E_0$ *is finite*  *iff*  $\theta _0 (\cdot )$ *is bounded.*

## Concluding remarks

The main aim of this letter is to show that the influence of a local magnetic field on the Welsh eigenvalues depends nontrivially on the magnetic flux. In order to make the exposition easy, we focused on the simple setting with radial $\delta$ potentials and an Aharonov–Bohm field, however, we are convinced that the conclusions extend to other potentials and other magnetic field profiles, as long as the radial symmetry and periodicity are preserved. This could be a subject of further investigation, as well as the remaining spectral properties of the present simple model such as the eigenvalue accumulation for $\alpha \in (0,\alpha _{\mathrm {crit}})$ or (non)existence of eigenvalues for $\alpha \in [\alpha _{\mathrm {crit}}, \frac{1}{2} )$ and an arbitrary $\beta \ne 0$. It would be also interesting to revisit from the present point of view situations in which the radially periodic interaction is of a purely magnetic type with zero total flux [12].
